# WENDI: A tool for finding non-obvious relationships between compounds and biological properties, genes, diseases and scholarly publications

**DOI:** 10.1186/1758-2946-2-6

**Published:** 2010-08-20

**Authors:** Qian Zhu, Michael S Lajiness, Ying Ding, David J Wild

**Affiliations:** 1 School of Informatics and Computing, Indiana University, Bloomington, IN, USA; 2 Eli Lilly and Company, Indianapolis, IN, USA; 3 School of Library & Information Science, Indiana University, Bloomington, IN, USA

## Abstract

**Background:**

In recent years, there has been a huge increase in the amount of publicly-available and proprietary information pertinent to drug discovery. However, there is a distinct lack of data mining tools available to harness this information, and in particular for knowledge discovery across multiple information sources. At Indiana University we have an ongoing project with Eli Lilly to develop web-service based tools for integrative mining of chemical and biological information. In this paper, we report on the first of these tools, called WENDI (Web Engine for Non-obvious Drug Information) that attempts to find non-obvious relationships between a query compound and scholarly publications, biological properties, genes and diseases using multiple information sources.

**Results:**

We have created an aggregate web service that takes a query compound as input, calls multiple web services for computation and database search, and returns an XML file that aggregates this information. We have also developed a client application that provides an easy-to-use interface to this web service. Both the service and client are publicly available.

**Conclusions:**

Initial testing indicates this tool is useful in identifying potential biological applications of compounds that are not obvious, and in identifying corroborating and conflicting information from multiple sources. We encourage feedback on the tool to help us refine it further. We are now developing further tools based on this model.

## Background

In common with most scientific disciplines, there has in the last few years been a huge increase in the amount of publicly-available and proprietary information pertinent to drug discovery, owing to a variety of factors including improvements in experimental technologies (High Throughput Screening, Microarray Assays, etc), improvements in computer technologies (particularly the Web), funded "grand challenge" projects (such as the Human Genome Project), an imperative to find more treatments for more diseases in an aging population, and various cultural shifts. This has been dubbed data overload [1] Significant effort has therefore been put into the development of computational methods for exploiting this information for drug discovery, particularly through the fields of Bioinformatics and Cheminformatics. Of particular note are the provision of large-scale chemical and biological databases, such as PubChem [2], ChemSpider [3], the PDB [4], and KEGG [5], which house information about massive numbers of compounds, proteins, sequences, assays and pathways; the development of predictive models for biological activity and other biological endpoints; data mining of chemical and biological data points; the availability of journal articles in electronic form, and associated indexing (such as in PubMed) and text mining of their content. Further, we are seeing an unprecedented amount of linking of information resources, for instance with Bio2RDF [6], Linking Open Drug Data [7] and manual linking of database entries.

One of the next great challenges is how we can use all of this information together in an intelligent way, in an *integrative *fashion [8]. We can think of all these information resources as pieces of a jigsaw, which in their own right give us useful insights, but to get the full picture requires the pieces to be put together in the right fashion. We thus not only need to aggregate the information, but we also need to be able to data mine it in an integrative fashion. There are a number of technologies that are becoming available that assist with this: in particular, web services and Cyberinfrastructure [9] allow straightforward, standardized interfaces to a variety of data sources and Semantic Web languages such as XML, OWL and RDF permit the aggregation of data, and representation of meaning and relationships in the data respectively.

At Indiana University, we are tackling this problem from several angles. We recently developed a Cyberinfrastructure for cheminformatics, called *ChemBioGrid*, which has made a multitude of databases and computational tools freely available for the first time to the academic community in a web service framework [10]. Of particular import, we have been able to successfully index chemical structures in the abstracts of large numbers of scholarly publications through a collaboration with the Murray Rust group at Cambridge. The infrastructure has spurred the development of several important client applications, including PubChemSR [11], and the application of Web 2.0 style "mashups" using userscripts for a variety of life-science applications [12]. We are continuing to support and further develop this infrastructure.

With this infrastructure in place, we have investigated a variety of strategies for integrating the chemical and biological data from different sources in the infrastructure, in particular of (i) the application of data mining techniques to chemical structure, biological activity and gene expression data in an integrated fashion [13], (ii) the development of a generalizable four layer model (storage, interface, aggregation and smart client) for integrative data mining and knowledge discovery [14], and (iii) aggregation of web services into automatically generated and ranked workflows [15]. We are now investigating methods for applying these techniques on a larger scale, particularly to be able to extract knowledge from large volumes of chemical and biological data that would not be found by searching single sources, and to be able to use multiple independent sources to corroborate or contradict hypotheses. To do this, we are employing two key technologies: *aggregate web services *which call multiple "atomic" web services and aggregate the results, and Semantic Web languages for the representation of integrated data.

In this paper we describe one of the first products of this work, a tool called *WENDI *(Web Engine for Non-obvious Drug Information) that is designed to tackle a specific question: given a chemical compound of interest, how can we probe the potential biological properties of the compound using predictive models, databases, and the scholarly literature? In particular, how can we find *non-obvious *relationships between the compound and assays, genes, and diseases, that cross over different types of data source? We present WENDI as a tool for aggregating information related to a compound to allow these kinds of relationships to be identified.

Of course, the power of this kind of integration comes from identifying truly non-obvious but yet real relationships between these entities. Our aim in this work is to allow a rapid differentation between known relationships (i.e. those which a scientist with a reasonable understanding of the literature in a field could be expected to already know), and unknown relationships (those which could not be found in literature closely associated with a field, or not part of the 'art' of the field). There is clearly some fuzziness in this, and this makes evaluation of a tool like WENDI for non-obviousness difficult. However, we do present it as a useful tool based on qualitative feedback from existing users, and we are currently devising ways of a more quantitative evaluation (as described in the concluding section).

## Implementation

### 1. Overall architecture

**Figure 1:**
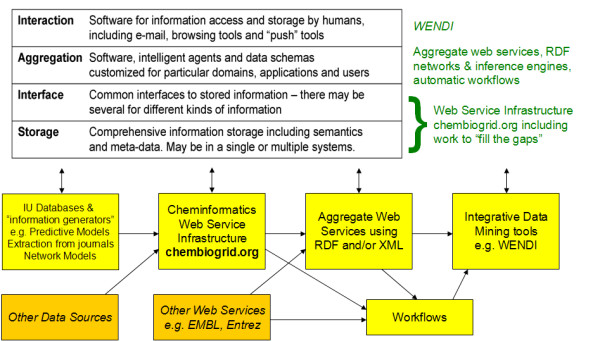
**Overall architecture of storage, interface, aggregation and interaction layers employed in WENDI**. Each layer can be accessed directly, or by higher layers.

We have since extended the *ChemBioGrid *infrastructure to be the primary data source for WENDI. Additionally, for WENDI we have introduced the idea of *aggregate web services *that call multiple individual, or *atomic*, web services and aggregate the results from these services in XML. For example, the main web service used by WENDI takes as input a SMILES string representing a compound of interest, and outputs an XML file of information about the compound aggregated by calling multiple web services. This XML file can then be parsed by an intelligent client to extract information pertinent to compound properties. The overall architecture uses a four layer approach which we described previously [14] that includes storage, interface, aggregation and smart interface layers (see Figure 1). The storage and interface layers are implemented using the Web Service Infrastructure, and our initial work developing aggregate web services and smart clients comprises the work described here.

Web services either follow the Simple Object Access Protocol (SOAP) standard [16] or REpresentational State Transfer (RESTful) approach [17], the latter of which are often better integrated with Hypertext Transfer Protocol (HTTP) than SOAP-based services. Whilst we have both kinds of web service in operation, we primarily use REST service. For example, we have created a 3D similarity searching Web Service is based on our local PubChem 3D database which stores 3D structures [18] and 12 distance moments [19] for all the compounds in the PubChem database. This service is called by the WENDI web service.

Our SOAP-based services are deployed in a in Tomcat 5.5 application container, which allows us to maintain these services easily and provides a high level of integration with our development environments, and with the service developed by Java 1.6.0. Our Web service layer is handled by the AXIS libraries 1.6 [20], which accept a SOAP message, decode it to extract the relevant function arguments, call the appropriate Web service classes, and finally encode the return value into a SOAP document for return to the client. Our Web service is published as WSDL [21] which is an XML-based standard for describing Web services and their parameters. Increasingly, we are converting our services to REST for even easier maintenance and access. A list of some of our atomic web services can be found on the web [22]

### 2. Database Services

Our infrastructure contains a large number of compound-related databases, including mirrors of existing databases (such as PubChem), databases derived from these (such as 3D structures of PubChem compounds), and completely new databases (particularly those derived from the literature). Our databases are housed on a Linux server running the PostgreSQL database system, with gNova CHORD [23] installed to allow chemical structure searching and 2D similarity searching through the generation of fingerprints. Mirrored databases are updated monthly. By housing the databases in a homogenous environment, it is easy to perform searches that cross multiple databases using single SQL queries, and to routinely expose the databases with web service interfaces. The following databases are used in the WENDI system:

#### PubChem Compound

A mirror of the PubChem Compound database, containing compound ID's (CIDs), InChI, SMILES, compound properties, and 166-key MACCS-style fingerprints [24] generated by the gNova CHORD system.

#### PubChem Bioassay

A mirror of the PubChem Bioassay database containing AIDs (assay ID's), CIDs of compounds tested, and bioassay outcomes and scores

#### PubChem BioDesc

Descriptions of all PubChem bioassays

#### Pub3D

A similarity-searchable database of minimized 3D structures for PubChem compounds

#### Drugbank

A mirror of the DrugBank dataset [25] containing CID's (mapping to PubChem), DBID's (Drugbank ID's), drug names, SMILES, usage descriptions, and 166-key fingerprints. The database contains nearly 4800 drug entries including >1,350 FDA-approved small molecule drugs, 123 FDA-approved biotech (protein/peptide) drugs, 71 nutraceuticals and >3,243 experimental drugs.

#### MRTD

An implementation of the Maximum Recommended Therapeutic Dose (MRTD) set [26] including name, SMILES, and 166-key fingerprints. The database contains 1,220 current prescription drugs available in SMILES format from the FDA Web site.

#### Medline Chemically-aware Publications Database

PubMed IDs of papers indexed in Medline[27], with SMILES of chemical structures (from the title and abstract) extracted using the Oscar3 program [28]

#### Phenopred

a matrix of predictions of gene-disease relationships based on known relationships mined from the literature and machine learning predictions [29].

#### Comparative Toxicogenomics Database (CTD)

cross-species chemical-gene/target interactions and chemical-disease relationships derived from experimental sets and the literature [30].

#### HuGEpedia

an encyclopedia of human genetic variation in health and disease [31].

#### ChEMBL

a database of bioactive drug-like small molecules, containing 2-D structures, calculated properties (e.g. logP, Molecular Weight, Lipinski Parameters, etc.) and abstracted bioactivities (e.g. binding constants, pharmacology and ADMET data) [32].

2D Tanimoto similarity searching of these datasets is made available by the gNova CHORD *tanimoto *function applied to the 2D *public 166 keys*, an implementation of the popular MACCS keys. Without indexing, it runs very effectively for a single query or on a small dataset, but the speed reduces significantly for large datasets. We have 56,911,891 compounds in our PubChem Compound table as the time of writing. To speed up the searching, we implemented a method described by Swamidass & Baldi to reduce the subset of molecules that need to be searched in similarity calculations [33]. The method uses simple bounds on similarity that can be applied when a similarity threshold is used (given two fingerprints A and B, and a threshold t, we can calculate a maximum similarity between the fingerprints as min (a,b)/(a+b-min (a/b)), where a and b are the number of bits set in A and B respectively).

In addition to 2D similarity searching, 3D similarity searching is provided on Pub3D database using 12-dimensional molecular shape descriptors [20] calculated for our Pub3D database of 3D minimized structures of PubChem compounds. Similarity to a query is calculated using Euclidean Distance. We use PostgreSQL to store all these 12D vectors for all compounds, with the CUBE type [34] extension.

### 3. Prediction services

We have made available a variety of predictions through our web service framework, particularly:

• **Tumor cell line predictions**. We created 40 Random Forest models for prediction of human tumor cell line inhibition, trained using data from the NCI Developmental Therapeutics Program Human Tumor Cell Lines [13]. These predictions output a probability of activity for a compound (0-1).

• **Toxicity prediction**. We implemented a special modified Web service implementation of ToxTree [35] for prediction of toxic effects

• **Gene-disease relationships**. We have implemented a table of predictions of gene-disease relationships extracted from the PhenoPred tool developed at Indiana University [29]. Also we employed the CTD and HuGEpedia data to expore gene-disease relationships,

### 4. Aggregate web service and client

We have created a main WENDI aggregate web service, and a web-based client that employs the web service. The web service takes a query SMILES string as input (through a SOAP or REST interface), and calls a variety of web services and database searches using the query. Results are returned as an aggregate XML file with sections delineated according to the atomic web service that was called. Additional XML tags are added by the web service, in particular, Gene Ontology terms in the PubChem Bioassay descriptions, Drug descriptions (from Drugbank) and paper titles and abstracts, are extracted and tagged with Gene Ontology ID's (GOID's). These permit associations to be made between genes and assays, drugs and papers.

The client permits the user to input a SMILES string, or to draw a structure in using the JME editor [36], and then uses JSP (Java Server Pages) to submit the query request to the web service and display and parse the XML results, and JavaScript to handle the XML file as the response return back from the server side. The layer between request submitted by the client and response returned back from the server is effected using AJAX (shorthand for Asynchronous JavaScript + XML) technology. With Ajax, web applications can retrieve data from the server asynchronously without interfering with the display and behavior of an existing page.

The primary way that the databases are employed in WENDI is through similarity searching: finding compounds in the databases that are similar to the query, which have some known property: for example, we retrieve compounds that are similar (>0.85 Tanimoto) to a query molecule that are active in a given bioassay, are known drugs, or are referenced in a journal article. Based on the similar property principle [37] we can assume that these molecules are likely to have similar properties to the query compound, thus be of interest in understanding the potential properties of the query.

The WENDI interface is organized into six major sections:

*Predictive models results *presents the predicted probability of activity of the compound in 40 Human Tumor Cell line assays, organized by panel type (renal, non-small cell lung, breast, colon, etc) and color coded according to probability of activity (red for > = 0.7, yellow for > = 0.6 and <0.7, and grey for <0.6). Confusion metrics are also presented to allow the validity of these models to be assessed. Also presented are the results of a ToxTree analysis, particularly the classification according to Cramer rules [38] and a breakdown of presence or absence of known toxic fragments.

*Activities of similar compounds *presents a list of similar compounds (Tanimoto similarity values given) in PubChem that have been tested in bioassays, and shown to be either active. A link to the bioassay along with the bioassay name is given, and an additional column uses the extraction of Gene Ontology terms from the bioassay description along with the PhenoPred predictions of gene-disease relationships to list possible related diseases. The DrugBank and MRTD sets are also similarity searched with the results presented in a similar fashion; in the case of DrugBank, drug usage descriptions are given along with predictions of diseases extracted in a similar way to the PubChem section

*Similar compounds from chemogenomics data *presents a list of similar compounds (Tanimoto similarity values given) from CTD, ChEMBL data that include the relationships with compounds and genes/diseases.

*Similar compounds from Systems data *presents a list of similar compounds (Tanimoto similarity values given) from KEGG data that include the relationships with compounds and Pathways/Enzymes.

*Similar compounds in the literature *lists journal articles in Medline where the title or abstract contains compounds with a Tanimoto similarity >0.85 to the query. Links are given to the Journal articles

*Inactivities of similar compounds *presents the same informations as *Activities of similar compounds *sections, except for all of the similar PubChem compounds found that have been tested in bioassays and shown to be inactive.

Finally, a link is given to the raw XML file, and PDF file for download.

## Results

**Figure 2:**
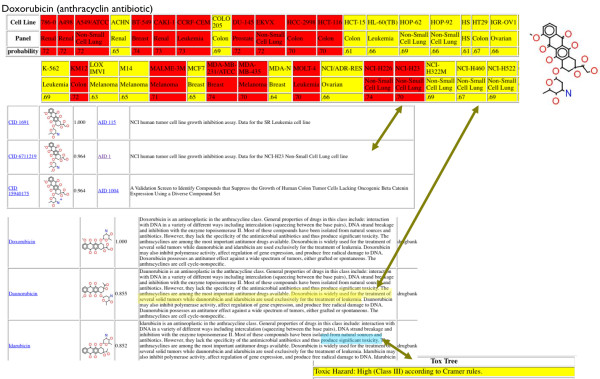
**Screenshot of the results returned from WENDI for Doxorubicin**.

On submission of a query, WENDI generally returns results within a minute. We have tested WENDI with a variety of query compounds with known biological activities, one of them is described below. It can be simply tested by the reader by visiting the WENDI site. As an example, a screenshot of the first results returned for Doxorubicin are shown in Figure 2.

Doxorubicin is an anthracyclin antibiotic that is used primarily as a nonspecific tumor inhibitor (including cancers of the bladder, breast, stomach, lung, ovaries, thyroid, along with soft tissue sarcoma and multiple myeloma). The mechanism of action is not fully understood, although it is thought to be a DNA intercalator.

**Figure 3:**
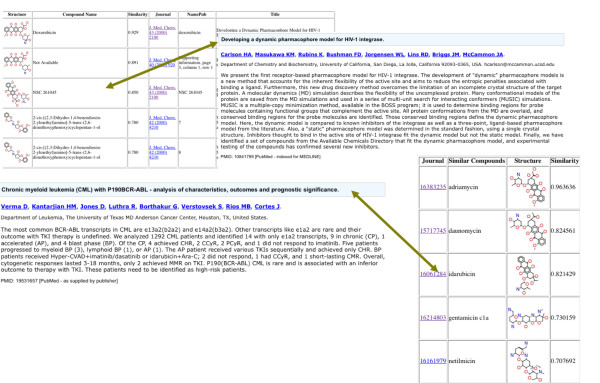
**Screenshot of the insights from the literature returned from WENDI for Doxorubicin**.

WENDI identifies several corroborating pieces of evidence for the biological actions of Doxorubicin. In particular, it (i) predicts that the compound has a high probability of activity in all but one of the tumor cell line screens (red) and a medium probability in HCT-15 (colon cancer); (ii) predicts that the compounds has toxic effects by our Toxicity prediction service, corroborated by descriptions from DrugBank; (iii) identifies specific tumor-related bioassays in which compounds similar to Doxorubicin (and identical to it) were found to be active (in particular, many similar compounds were found to be active in NCI Tumor Cell Line screens, corroborating the predictions of activity); (iv) identifies a wide variety of assays in which compounds similar to Doxorubicin are inactive; (v) identifies several similar drugs to Doxorubicin (Epirubcin, Daunorubicin, Idarubicin) along with descriptions corroborating the nonspecific anti-tumor activity; (vi) identifies numerous publications linking Doxorubicin and related compounds to a variety of tumor activities (Figure 3)

A chemical compounds recently submitted to PubChem, but not collected in our database yet, were also used as queries for WENDI. The results and some interpretations are given in the Table 1 and more results of other compounds tested by WENDI are shown in Table 2.

**Table 1:** Query compounds and related biological activities retrieved from WENDI

Query CID	44246308	44246315	44247545
Reported activities	weak activity against Sortase-A (SrtA), an antimicrobial target	tested and shown negative for activity against DNA polymerase alpha and beta	None

Tumor Cell Line Predictive models	50-60% probability of activity in breast, renal, prostate, HS, ovarian, leukemia, melanoma, non-small cell lung; otherwise <50% probability	50-60% probability of activity in renal, leukemia, non-small cell lung, colon, melanoma; otherwise <50% probability	<50% probability for all tumor cell lines

Bioassay activities & gene relationships of similar compounds	highly similar molecules found to be antagonists of GPCR GPR7 (associated with feeding behavior, obesity and inflammatory pain); CYP2C9 (metabolizes NSAIDS and sulfonylureas); inhibition of Non small-cell lung cancer (NCI HOP-18) and supression of colon tumors; inhibition of HIV-1 RNase H	similar molecules are shown active in CYP3A4 confirmation assay (important in drug metabolism); CYP2C9 (metabolizes NSAIDS and sulfonlyureas); BAP1 inhibition (tumor suppressor involved in breast cancer BRCA1); probes of Alpha-Synuclein 5'UTR (related to Parkinsons disease); FPR (GPCR involved in chemotaxis); antibacterial activity (Mycobacterium tuberculosis and VIM-2 metallo-beta-lactamase)	similar compounds show activity in CYP2C19 (metabolism of antiepeleptics and protein-pump inhibitors); agonist of M1 muscarinic receptor (associated with Alzheimer's and antipsychotics); Estrogen receptor alpha coactivator binding inhibitors (breast cancer association);

Bioassay inactives of similar compounds	many highly similar compounds (including one with a nominal 1.0 similarity) show inactive in RNase H screen (AID-372)	similar molecules inactive for HIV inhibition; inhibition of breast tumors (BRCT:pBACH1 of BRCA1); hERG inhibition; HIV-1 RNase H inhibition; 14-3-3 protein interaction modulators; antibacterial (Mycobacterium tuberculosis); FKBP12 immunosupressant;	similar compounds inactive for Cdc25B catalytic domain protein tyrosine phosphatase; beta-glucocerebrosidase inhibitors (linked with Gaucher disease); 14-3-3- protein interaction modulation; hERG blockers of proarrythmic agents

CTD gene relationships of similar compounds	similar compounds show link with use of anti-inflammatory drugs (NSAIDS) in carcinomas; CYP2C9;	similar compounds linked with Gilbert disease; adenoma; use of anti-inflammatory drugs (NSAIDS) in carinomas; coronary arterial protection; colorectal neoplasms (tumors)	None

Activities of similar marketed drugs	None	None	None

Insights from similar compounds in journal articles (MEDLINE)	None	Intricatin, a similar isofavonoid, is shown to be antimutagenic; Claussequinone has anti-inflammatory activity	None

Interpretation	Some evidence for anti-inflammatory activity (particularly related to tumors) and CYP2C9 inhibition; mixed evidence on generalized anti-tumor activity and inhibition of HIV-1 RNase H44	Generalized, nonspecific activity, although may be worth investigating for anti-tumor activity particularly colon cancer.	None

**Table 2:** More Query compounds and related biological activities retrieved from WENDI

Query CID	44246407	44246344
Reported activities	Inhibitor/activator of human alpha glucosidase	None

Tumor Cell Line Predictive models	50-60% probability of activity in melanoma, leukemia, otherwise <50% probability	Strong prediction (>70% probability) of activity in prostate, colon, non-small cell lung, breast, malanoma, leukemia, ovarian cancers. 50-60% probability in all other cell lines.

Bioassay activities & gene relationships of similar compounds	similar compound shows active as an inhibitor of MEK-5 Kinase 2 mutant	Similar compounds show active in NCI ovarian cancer cell line (IGROV1), breast cancer cell line (MB-435); non small cell lung cancer (H23); MLPCN Alpha-synuclein 5'UTR binding activation (Parkinson's disease); Leishmania promastigote inhibition; NCI yeast anticancer screen; RAM inhibition (STAT3);

Bioassay inactives of similar compounds	similar compounds show inactive in SIP3 antagonists assay, hERG blockers of proarrythmic agents. and 14-3-3- protein interaction modulation	similar compounds inactive in RNase H inhibition, NCI non small cell lung cancer (H23) and Leukemia (L1210); NCI yeast anticancer screen; 14-3-3 protein interaction modulators; SIP3 antagonists

CTD gene relationships of similar compounds	A similar compound associated with adenomatous polyposis	Similar compounds associated with Alzheimer's disease

Activities of similar marketed drugs	None	None

Insights from similar compounds in journal articles (MEDLINE)	None	None

Interpretation	None	None

## Conclusion

In this paper, we present a integrative data mining tool for drug discovery using aggregate web services. WENDI aims to build a full picture of potential biological activities of a chemical compound through the aggregation of data from web services that represent diverse multiple sources (including predictive models, databases and journal articles). WENDI allows the identification of corroborating or conflicting information: for instance, a compound might be predicted active in a breast cancer cell line, and similar compounds might show active in a PubChem BioAssay related to breast cancer, or be co-located in a paper abstract with a breast cancer related gene. We are now deveoping a next generation of tools based on WENDI and our recent Chem2Bio2RDF system [39] for exploring inferred relationships between compounds and diseases, genes, pathways using Semantic Web technologies including ontologies and RDF. We are also devising ways of quantitatively evaluating the extent to which WENDI truly identifies 'non-obvious' kinds of relationship, including using a corpus of literature in the field as the baseline for the 'obvious' relationships, as well as courting specific case studies from users for qualitative analysis.

## Availability and requirements

Project name: WENDI (Web Engine for Non-obvious Drug Information)

• Project home page: https: https://cheminfov.informatics.indiana.edu:8443/WENDI_PUBLIC/WENDI.jsp

• Operating system(s): Platform independent

• Programming language: Java

• Other requirements: Java browser-embedded plugin

• License: None. Any restrictions to use by non-academics: None

## Competing interests

The authors declare that they have no competing interests.

## Authors' contributions

QZ carried out the whole implementation of WENDI, supervised by MSL and DJW. DJW made the examples in the result section, MSL and DJW modified this paper from the draft written by QZ. All authors have read and approved the final version of the manuscript.
